# Audit of Mental Health Assessment in Leicester's Paediatric Emergency Department

**DOI:** 10.1192/bjo.2026.11428

**Published:** 2026-06-30

**Authors:** Alireza Majlessi, Samantha Jones, Jessica Gosney

**Affiliations:** 1 Leicestershire Partnership NHS Trust, Leicester, United Kingdom; 2 University Hospitals of Leicester NHS Trust, Leicester, United Kingdom

## Abstract

**Aims:**

This retrospective quality improvement project audited how well children and young people (CYP) who presented with mental health concerns to Leicester's Paediatric Emergency Department (PED) were being assessed. During the audit period, Leicester's PED had two digital forms available to be filled out by the triaging clinical staff. One of the forms was titled the emergency department (ED) mental health risk assessment tool which determines what is needed to keep CYP safe while they wait to be seen, and a second form titled the ED mental health form which includes mental state examination and a dynamic priority score (DPS). Completion of these tools fits in with meeting the requirements of the NICE Guidance (NG225).

**Methods:**

This was a retrospective audit of 59 patients who presented to the Leicester PED with acute mental health concerns in September 2024. Data collection took place onNerveCentre, which is the digital software Leicester's ED uses for the documentation of clinical activities.

The following data was collected:

1. Percentage of total completed mental health risk assessments using the assessment tool.

2. Percentage of ED mental health forms which were opened/started by staff.

3. Percentage of ED mental health forms which had a completed mental state examination.

4. Percentage of ED mental health forms which had a DPS score assigned.

**Results:**

1. 11.9% (7/59) patients had a completed ED mental health risk assessment using the assessment tool.

2. 62.7% (37/59) patients had an ED mental health form opened/started by staff.

3. 43.2% (16/37) of the ED mental health forms which were opened had a completed mental state examination.

4. 78.4% (29/37) of the ED mental health forms which were opened had a DPS score assigned to signify the level of risk.

The audit also revealed that the tool may not be as suitable for CYP (mentions of capacity rather than competence, high-risk outcome suggests rapid tranquillisation). Additionally, there is overlap between the tool and the form, which may be contributing to the lower completion rates of the tool.

**Conclusion:**

This audit revealed that Leicester PED's initial assessment of CYP presenting with acute mental health concerns needs to be reviewed, with enhancements made to make the tools more applicable for CYP. Revamped tools can then be further embedded with education and training amongst staff to increase the department's adherence to the NICE Guidance (NG225).

